# Coccidioides-Induced Pyopneumothorax in an Immunocompetent Patient

**DOI:** 10.7759/cureus.39782

**Published:** 2023-05-31

**Authors:** Seyed Khalafi, Michael J Brockman, Fatma Dihowm

**Affiliations:** 1 Medicine, Paul L. Foster School of Medicine, El Paso, USA; 2 Internal Medicine, Paul L. Foster School of Medicine, El Paso, USA; 3 Internal Medicine, Texas Tech University Health Sciences Center, El Paso, USA

**Keywords:** pyopneumothorax, cavitary lung lesion, coccidioides, immunocompetent, pneumonia, coccidioidomycosis

## Abstract

Coccidioidomycosis is a rare infection caused by the dimorphic fungi *Coccidiodes immitus* or *Coccidioides posadasii*. This fungal infection is very common in the American Southwest as well as northern Mexico. Though the fungus is ubiquitous, symptomatic coccidioidomycosis usually occurs in the elderly or immunocompromised. This case report discusses a unique instance of an immunocompetent 29-year-old male without any notable past medical history who was found to have a coccidioidal cavitary lung lesion with concomitant pyopneumothorax.

## Introduction

Coccidioidomycosis is a pulmonary fungal infection caused by the pervasive dimorphic fungi Coccidiodes immitus and/or Coccidioides posadasii. The fungi mostly reside in the soil of desert climate areas of the southwest United States, Mexico, and parts of Central and South America [1,2]. The fungus spreads via spores, which are broken off during rainstorms, and swept through the air via strong winds. The spores, being very hearty in nature, can penetrate deep into the pulmonary tract of individuals who inhale the spores. Though ubiquitous, only 40% of infected individuals show symptoms, which can often be related to immunosuppressant comorbidities [3,4]. The clinical manifestations of coccidioidomycosis consist of primary pulmonary infections or disseminated disease. Severe infection with dissemination is a rare complication, accounting for 1% to 3% of all cases [5,6]. In endemic areas, coccidioidomycosis can account for 25% of all community-acquired pneumonia [7].

Due to the westward spread of the American population, as well as the ease of travel in the United States, the incidence of coccidioidomycosis is increasing. According to the CDC, the rate has increased from 5.3 per 100,000 people in 1998 to 42.6 per 100,000 in 2011 [2]. A California infectious disease study showed an increase from 2.4 to 18.8 cases per 100,000 people from 2000 to 2018 [8]. The body of literature supporting the endemic disease’s expansion attributed it to rapid desert developments, whether agricultural or residential, in areas such as Phoenix, Arizona, or Southern California [9].

In this case report, we present a 29-year-old male who presented with shortness of breath and was found to have a pyopneumothorax with a cavitary coccidioidomycosis infection and pleural effusion.

## Case presentation

A 29-year-old Hispanic male with no pertinent past medical history presented with a four-day history of shortness of breath. The patient worked as a boxing personal trainer and mixed martial artist with repeated chest trauma. What began as strenuous shoulder exercises transformed into extreme shoulder and chest discomfort. Assuming it was musculoskeletal shoulder pain, he received a dexamethasone joint injection, which improved his pain but did not resolve his shortness of breath. Two days later, the patient developed a 104° Fahrenheit fever that wasn’t alleviated by acetaminophen. Given his continued worsening shortness of breath, he presented to the emergency room.

On presentation, the patient was afebrile with a temperature of 36.6° Celsius, had tachycardia (103 beats/min), normotensive (127/68 mm of Hg), non-tachypneic (18 breaths/minute), and had a saturation of peripheral oxygen (SpO2) of 89% on room air. On physical exam, the patient was alert and oriented. The patient’s neck was supple with the trachea midline. There were clear breath sounds on the left side and absent breath sounds on the right lung. He had a normal heart rate, a regular rhythm with no murmurs appreciated, and no focal neurological deficits. Chest X-ray revealed a large-volume right-sided pneumothorax with the complete collapse of the right lung and a small to moderate volume of right-sided pleural effusion. The patient subsequently underwent chest tube placement with a Wayne catheter, producing 800 mL of chylous fluid with <20 mg/dL of glucose, 3,057 units/L of lactate dehydrogenase, and negative fluid cultures for bacteria or fungi.

On admission, a complete blood count panel showed normal leukocyte count, hemoglobin, hematocrit, and platelet count. A complete metabolic panel on admission also presented normal electrolytes and liver function. A urine drug screen was positive for cannabinoids, amphetamines, cocaine, and opiates. Concerned for immunosuppression, an HIV screen was done, which was negative. Selenium levels were also checked to rule out cardiomyopathy, which was within normal limits as well. 

**Figure 1:**
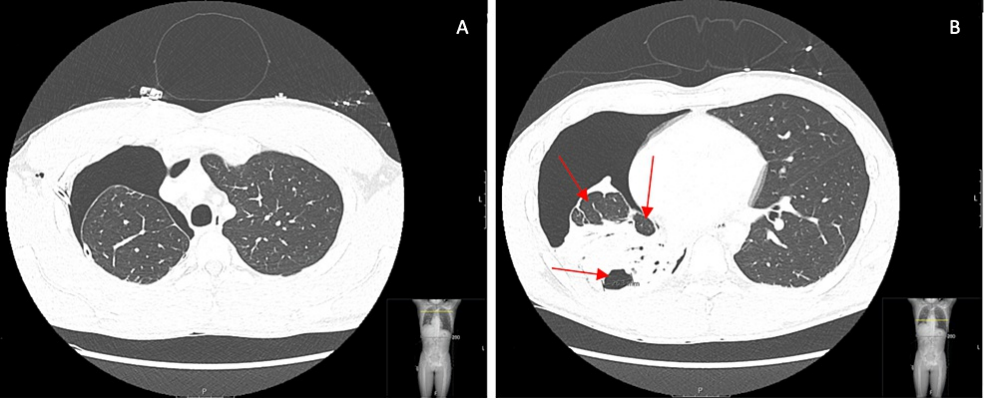
Initial chest CT with contrast of the upper (A) and lower (B) lungs in the axial view Observed is a large volume right pneumothorax with the collapse of the right lower lobe and subsegmental atelectatic changes of the right upper lobe. Red arrows point to persistent atelectasis of the right lower lobe with a parenchymal cystic lesion. Also observed was thickening and retraction of the visceral pleura with adjacent parenchymal fibrosis suggesting a trapped lung.

**Figure 2:**
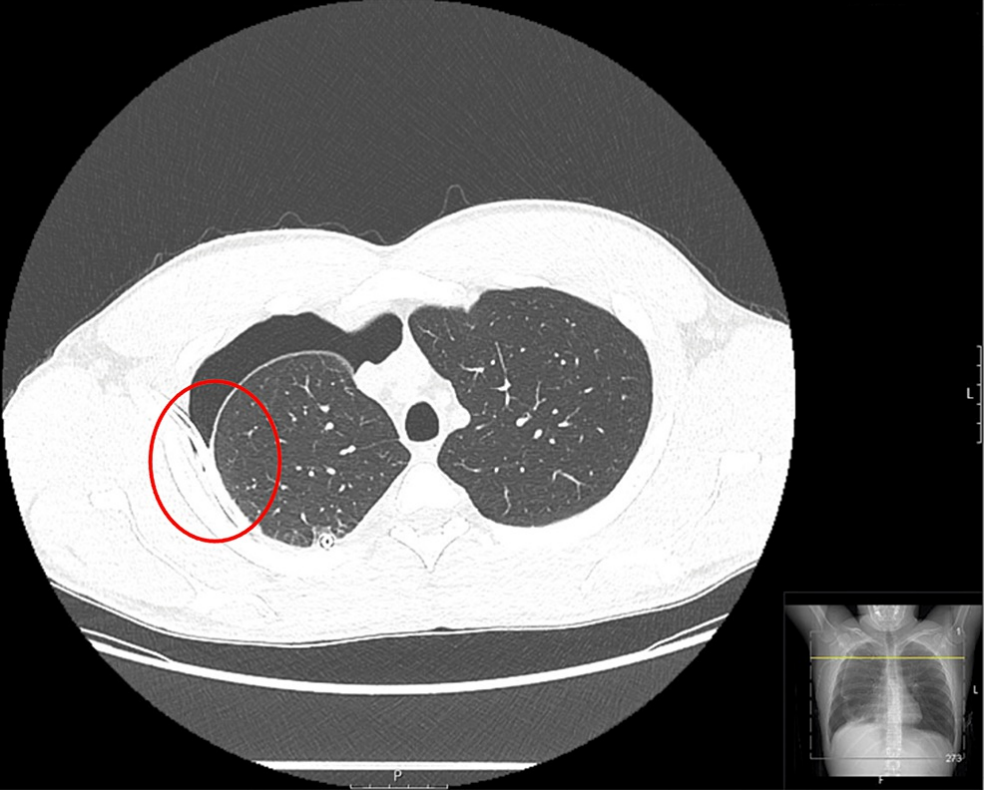
Chest CT without contrast of the upper lungs performed four days after initial CT Seen is a moderate to large, loculated hydropneumothorax that has improved. The right pleural effusion with locules of air is also indicated (red circle).

Once on the floor, a CT of the thorax with contrast revealed residual right-sided pyopneumothorax, persistent right lower lobe atelectasis with parenchymal cystic lesions, and thickening and retraction of the viscera pleura with adjacent parenchymal fibrosis (Figure 1). The patient was empirically started on ceftriaxone and clindamycin due to possible infectious causes of spontaneous pneumothorax. Given these findings, pulmonology was consulted, and the patient completed a bronchoscopy with bronchoalveolar lavage (BAL). The BAL culture found the right lower lobe cavitary lesion was due to Coccidiodes immitus. Infectious disease was consulted due to positive coccidioides results, and the patient was recommended oral fluconazole 400 mg daily for three months. A repeat chest CT four days later showed improvement of the pyopneumothorax and right pleural effusion (Figure 2).

**Figure 3:**
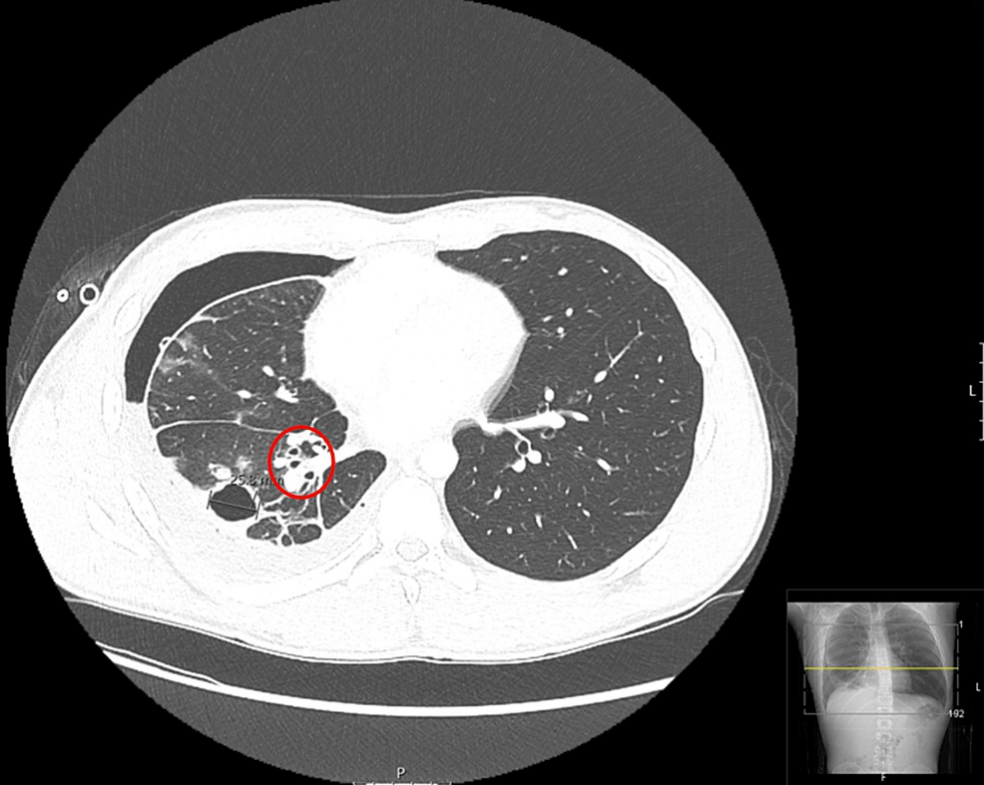
Chest CT with contrast of the lower lungs 11 days after initial CT Seen is a cystic lesion in the right lower lobe measuring 2.6 cm and slightly increased right pleural effusion with associated atelectasis. Mildly enlarged right hilar lymph nodes are also indicated (red circle).

Though the diagnosis was now clear, the patient continued to have residual pneumothorax and was placed on a non-rebreather mask on the sixth day of hospitalization. Serial chest X-rays and chest CT were utilized due to non-improving oxygen saturation. The diagnostic images revealed persistent right and left lower lobe consolidations despite chest tube suctioning and chronic scarring of the right lower lobe with thickening and retraction of the right visceral pleura, suspicious for a trapped lung (Figure 3).

Due to unresolved pyopneumothorax, an additional Wayne chest tube was placed by cardiothoracic surgery, however, proper positioning was unable to be attained. A chest X-ray performed four days later showed an interval increase in the pyopneumothorax, prompting cardiothoracic surgery to recommend video-assisted thoracoscopic surgery (VATS). The procedure was converted to an open right thoracotomy perioperatively, resulting in the re-expansion of the lung. The patient was then admitted to the surgical intensive care unit for closer observation. Once stable, the patient was de-escalated to the floor due to improving oxygen saturation and decreasing pyopneumothorax with the removal of chest tubes and discharged seven days later.

## Discussion

Our patient was found to have coccidioidomycosis, confirmed with antigen testing and a positive BAL. The diagnosis and treatment were complicated by a right-sided pyopneumothorax, pleural effusion, cystic lesions, and parenchymal fibrosis. With a refractory pneumothorax not reinflated with chest tube placement, challenges arose due to a trapped lung from extensive pleural inflammation, requiring a VATS procedure. While the patient did not have any known medical history, multiple confounders may have contributed to the difficulty of treatment, such as repetitive chest trauma and a history of drug use.

Previous studies have suggested some individuals are at an increased risk of developing symptomatic coccidioidomycosis and worse outcomes, including those with immunocompromised states, especially males aged >60; of African American, Hispanic, or Southeast Asian heritage; uncontrolled diabetes; living in endemic areas; smoking; and pregnant women [10-15]. Our patient did have some risk factors, including being male, Hispanic, living in an endemic area, and having a history of marijuana use.

Most patients with coccidioidomycosis present with pulmonary symptoms. The initial chest X-ray makes physicians suspicious for community-acquired pneumonia, prompting empiric antibiotic use [16]. Mild to moderate coccidioidal pneumonia usually clears up on its own, while complex infections can present in different ways. Complex presentations include pleural effusion, nodules, cavitations, and empyema [17]. Our patient presented with right lower lobe cavitary lesions, moderate volume pleural effusion, and extensive visceral pleural fibrosis causing trapped lung. Pleural effusion is a rare complication of coccidioidomycosis, observed in 5% to 15% of cases [18]. Furthermore, only 5% to 15% of pulmonary coccidioidomycosis subsequently develop cavitary lesions, with half of these presentations clearing radiographically on their own without treatment. Most cavitary lesions were also found in peripheral (32.8%) or subpleural (44.3%) areas of the lung [19,20]. Rupture of cavitary lesions and spontaneous pneumothorax is estimated to occur in 2% to 4.4% of cases [21]. Cavitary lesions in the periphery of the lung can cause empyema and extensive fibrothorax, resulting in trapped lung and very rare pyopneumathoraxes, which are more common in healthy athletic males, as is the case in our patient, and have a mortality rate of 15% [22,23].

Treatment of cavitary coccidioidomycosis requires early treatment to prevent pyopneumothorax or worse complications. According to the Infectious Diseases Society of America (IDSA) clinical practice guidelines for the treatment of asymptomatic cavitary coccidioidomycosis, the recommendation for uncomplicated patients is supportive treatment. As for symptomatic patients with ruptured coccidioidal cavities, the guidelines recommend the use of oral azole therapy; if contraindicated or more than two surgical procedures are required, the use of intravenous amphotericin B is warranted. Fluconazole has been used as a first-line treatment option for patients with mild to moderate pneumonia and non-life-threatening disseminated disease. Other antifungal drugs that have been used in studies include itraconazole, voriconazole, and posaconazole [4,16]. Our patient was treated with fluconazole, which was recommended by the expert opinion of the infectious disease department of the hospital. The IDSA clinical guidelines also recommend the VATS procedure in the management of ruptured coccidioidal cavities and pyopneumathoraxes rather than open thoracotomy, as was performed in our patient [4]. Complications after a VATS procedure include bronchopulmonary fistulas or prolonged air leaks [24].

## Conclusions

This case report presents a unique presentation of a common fungal infection. Given the patient’s lack of comorbidities, it is relatively rare to have such significant complications from coccidioidomycosis. The patient’s history of drug use and repetitive chest trauma could have played a factor in the disease's progression. A rare clinical presentation of cavitary lesions, pleural effusion, and pyopneumothorax was noted, which resulted in the patient having to undergo a VATS procedure. With serious complications that may arise in coccidioidomycosis infections, this case report sheds light on significant complications that can arise from a common albeit proliferating disease.

## References

[1] Thompson GR 3rd (2011). Pulmonary coccidioidomycosis. Semin Respir Crit Care Med.

[2] Crum NF (2022). Coccidioidomycosis: a contemporary review. Infect Dis Ther.

[3] Huang JY, Bristow B, Shafir S, Sorvillo F (2012). Coccidioidomycosis-associated deaths, United States, 1990-2008. Emerg Infect Dis.

[4] Galgiani JN, Ampel NM, Blair JE (2016). 2016 Infectious Diseases Society of America (IDSA) clinical practice guideline for the treatment of coccidioidomycosis. Clin Infect Dis.

[5] Bays DJ, Thompson GR, Reef S (2021). Natural history of disseminated coccidioidomycosis: examination of the Veterans Affairs-Armed Forces Database. Clin Infect Dis.

[6] Rosenstein NE, Emery KW, Werner SB (2001). Risk factors for severe pulmonary and disseminated coccidioidomycosis: Kern County, California, 1995-1996. Clin Infect Dis.

[7] Valdivia L, Nix D, Wright M (2006). Coccidioidomycosis as a common cause of community-acquired pneumonia. Emerg Infect Dis.

[8] Centers for Disease Control and Prevention (2013). Increase in reported coccidioidomycosis—United States, 1998-2011. MMWR Morb Mortal Wkly Rep.

[9] (2023). Epidemiological summary of coccidioidomycosis in California, 2018. https://www.cdph.ca.gov/Programs/CID/DCDC/CDPH%20Document%20Library/CocciEpiSummary2018.pdf.

[10] Adam RD, Elliott SP, Taljanovic MS (2009). The spectrum and presentation of disseminated coccidioidomycosis. Am J Med.

[11] Peterson CM, Schuppert K, Kelly PC, Pappagianis D (1993). Coccidioidomycosis and pregnancy. Obstet Gynecol Surv.

[12] Ampel NM, Dols CL, Galgiani JN (1993). Coccidioidomycosis during human immunodeficiency virus infection: results of a prospective study in a coccidioidal endemic area. Am J Med.

[13] Blair JE (2007). Coccidioidomycosis in patients who have undergone transplantation. Ann N Y Acad Sci.

[14] Brown J, Benedict K, Park BJ, Thompson GR 3rd (2013). Coccidioidomycosis: epidemiology. Clin Epidemiol.

[15] Guo X, Ruan Q, Jin J (2022). Disseminated coccidioidomycosis in immunocompetent patients in non-endemic areas: a case series and literature review. Eur J Clin Microbiol Infect Dis.

[16] Saubolle MA, McKellar PP, Sussland D (2007). Epidemiologic, clinical, and diagnostic aspects of coccidioidomycosis. J Clin Microbiol.

[17] Johnson RH, Sharma R, Kuran R, Fong I, Heidari A (2021). Coccidioidomycosis: a review. J Investig Med.

[18] Merchant M, Romero AO, Libke RD, Joseph J (2008). Pleural effusion in hospitalized patients with coccidioidomycosis. Respir Med.

[19] Panicker RR, Bartels HC, Gotway MB, Ampel NM, Buras MR, Lim ES, Blair JE (2021). Cavitary coccidioidomycosis: Impact of azole antifungal therapy. Med Mycol.

[20] Gadkowski LB, Stout JE (2008). Cavitary pulmonary disease. Clin Microbiol Rev.

[21] Collins J, Dy M, Clemens C, Faddoul D (2011). Not just a simple pneumothorax. Pediatr Infect Dis J.

[22] Galgiani J, Knox K, Rundbaken C, Siever J (2015). Common mistakes in managing pulmonary coccidioidomycosis. Southwest J Pulm Crit Care.

[23] Kanai O, Fujita K, Okamura M, Nakatani K, Mio T (2021). Afebrile tension pyopneumothorax due to anaerobic bacteria: fistula or gas production?. Respir Med Case Rep.

[24] Jaroszewski DE, Halabi WJ, Blair JE (2009). Surgery for pulmonary coccidioidomycosis: a 10-year experience. Ann Thorac Surg.

